# The dietary phytochemicals carnosic acid and sulforaphane regulate inflammatory markers in ulcerative colitis patient-derived colonoids

**DOI:** 10.3389/fphar.2025.1696576

**Published:** 2025-12-18

**Authors:** Rocío Rivera Rodríguez, Siri Sæterstad, Ann-Therese Chattergoon Ali, Linn-Karina M. Selvik, Ingunn Bakke, Torunn Bruland, Jeremy J. Johnson

**Affiliations:** 1 Department of Pharmaceutical Sciences, Retzky College of Pharmacy, University of Illinois Chicago, Chicago, IL, United States; 2 Department of Clinical and Molecular Medicine (IKOM), Faculty of Medicine and Health Sciences, NTNU - Norwegian University of Science and Technology, Trondheim, Norway; 3 Clinic of Laboratory Medicine, St. Olav’s University Hospital, Trondheim, Norway; 4 Department of Gastroenterology and Hepatology, Clinic of Medicine, St. Olav’s University Hospital, Trondheim, Norway; 5 Department of Pharmacy Practice, Retzky College of Pharmacy, University of Illinois Chicago, Chicago, IL, United States

**Keywords:** intestinal epithelial organoid, anti-inflammatory, inflammatory bowel disease, NRF2, NGAL, LCN2

## Abstract

**Introduction:**

The inflammatory bowel disease (IBD) ulcerative colitis (UC) is characterized by continuous inflammation of the colon with erosion and ulcers. Diagnosis typically occurs in patients between their late teens and mid-30s with no cure. Available therapeutics are efficient at controlling symptoms however, they have many serious adverse effects. Thus, additional therapies with limited adverse effects are needed to complement these drugs. In this study, we evaluated the anti-inflammatory potential of carnosic acid (CA), the most abundant diterpene in rosemary (*Salvia rosmarinus*) and sulforaphane (SFN), an isothiocyanate found in cruciferous vegetables.

**Methods:**

We used colonic epithelial organoids (colonoids) derived from non-IBD and UC patients as a physiologically relevant testing platform for both phytochemicals. These patient-derived colonoids are a representative model that recapitulates the parent epithelial tissue including its cellular composition and 3D structure. Moreover, we cultured the colonoids at 2% O_2_ to better approximate the low oxygen level (physioxia) observed in the colon crypts. To assess the effects of CA and SFN in the nuclear factor erythroid 2-related factor 2 (NRF2) and nuclear factor kappa-light-chain-enhancer of activated B cells (NF-κB) pathways, we studied modulation of inflammatory cytokines through a 40-plex chemokine assay and ELISA, as well as gene and protein expression of target genes with qPCR and western blot, respectively.

**Results:**

Through these techniques, we observed that CA and SFN decreased inflammatory markers and promoted NRF2 activity in patient-derived colonoids. Additionally, SFN and CA modulated the expression and secretion of the NF-κB promoted antibacterial peptide neutrophil gelatinase-associated lipocalin which is highly expressed in the inflamed colonic epithelium and has been suggested as a biomarker for active UC.

**Discussion:**

Together, the results validated the use of colonoids as a pharmacological testing platform for phytochemicals, and that CA and SFN promote NRF2 activation and decrease inflammation in a human physiologically relevant UC model.

## 1 Introduction

Inflammatory bowel diseases (IBD) are chronic inflammatory diseases with a complex and unknown etiology and consist of the major subtypes, Crohn’s disease and ulcerative colitis (UC) (Abraham and Cho, 2009). There are a variety of factors that influence disease progression, including genetics, environment, and intestinal microbiome. UC is characterized by continuous inflammation of the colon and development of ulcers and erosions (Colombel et al., 2019). Active disease over prolonged periods of time increase the probability of complications and development of extraintestinal manifestations (Colombel et al., 2019; Rubin et al., 2019). Proper and prolonged treatment is essential to increase quality of life as well as limit long term complications. Nonetheless, available therapies are linked to a variety of adverse effects and the gold standard, biologics, have a success rate of approximately 50% in IBD patients (Cai et al., 2021). Hence, there is a need for efficient novel therapies with low adverse effects that can be used alone or in combination with current drugs to decrease their dosage and/or make them more efficient (Cai et al., 2021; Click and Regueiro, 2019).

**FIGURE 1:**
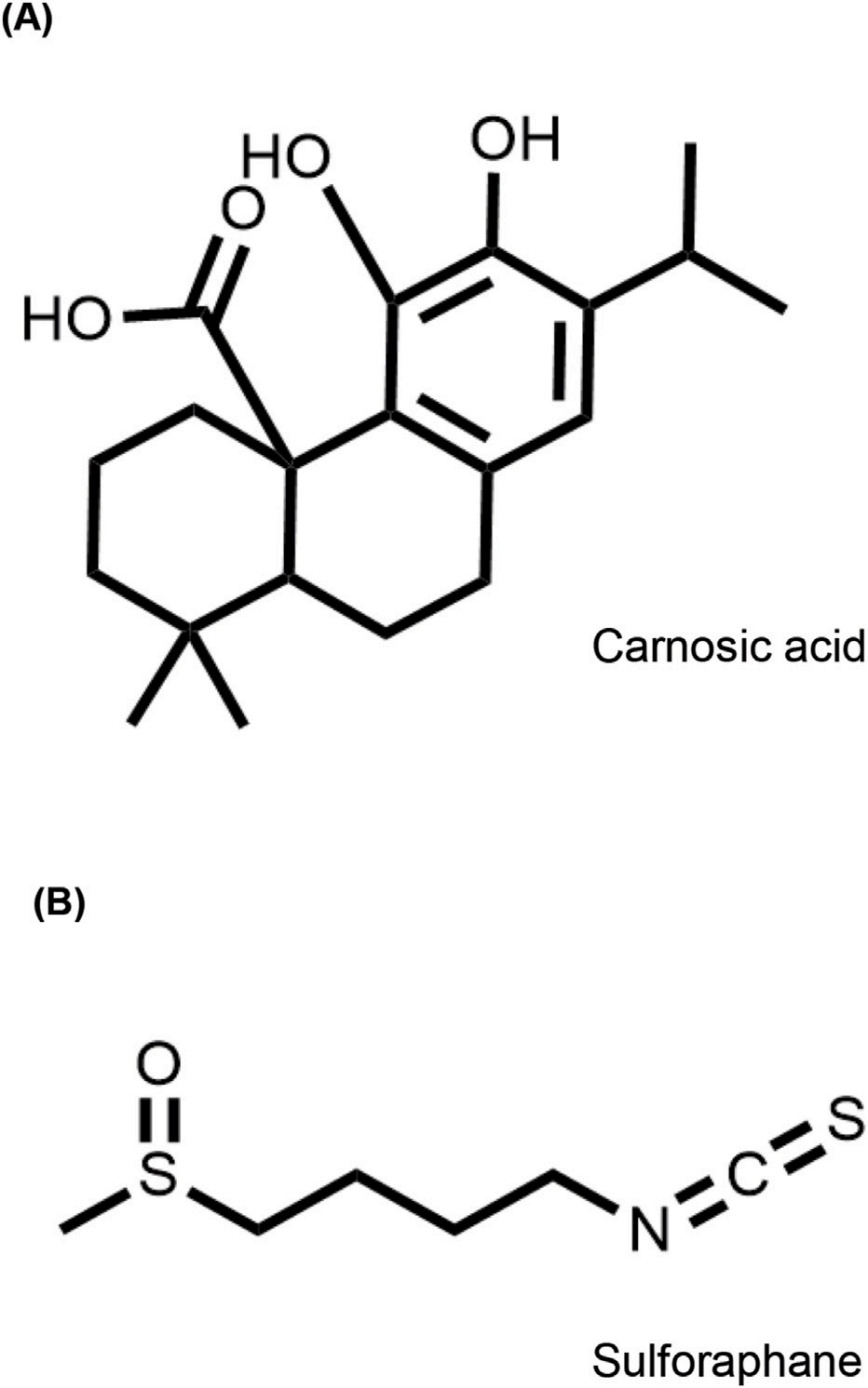
Molecular structures of **(A)** carnosic acid and **(B)** sulforaphane.

One area of interest regarding small molecules and modulation of inflammation in UC is natural products derived from a healthy diet. In this study, we focused on two small molecules, dietary phytochemicals; carnosic acid (CA), the most abundant diterpene in *Salvia rosmarinus* (rosemary) and sulforaphane (SFN), an isothiocyanate found in cruciferous vegetables (Figure 1) (Birtić et al., 2015; Campas-Baypoli et al., 2010). Oral administration of both compounds has been reported to alleviate inflammation and colitis symptoms in mice and rats with dextran sodium sulfate (DSS)-induced acute colitis (Alattar et al., 2022; Du et al., 2023; Wagner et al., 2013; Yang et al., 2017; Zhang et al., 2020; Zhou et al., 2024). Both compounds increased the activity of the nuclear erythroid 2-related factor 2 (NRF2), which is one of the most important endogenous antioxidant pathways. Oxidative stress is a key mediator of inflammation in many diseases, including UC, rapidly activating NRF2 to repress the major pro-inflammatory transcription factor, nuclear factor kappa-light-chain-enhancer of activated B cells (NF-ƙB), and promote transcription of antioxidant and detoxifying enzymes (Peng et al., 2023; Sahoo et al., 2023).

Neutrophil gelatinase-associated lipocalin (NGAL) is a siderophore-sequestering, anti-bacterial peptide produced by many cell types, including intestinal epithelium where it is upregulated by tissue stress and inflammation. Multiple studies have found a positive correlation with colitis severity, thus, its proposal as a UC biomarker (Thorsvik et al., 2018; Zollner et al., 2020). NGAL has shown multifaceted roles, e.g., neutrophil chemoattraction, stabilization of matrix metalloproteinase 9 (MMP-9) and promotion of epithelial regeneration, cell migration, and goblet cell maturation (Liu et al., 2019; Saha et al., 2017; Thorsvik et al., 2019; Yan et al., 2001), and there is no consensus on whether upregulation or downregulation is more advantageous (Liu et al., 2019; Singh et al., 2016a). Lipocalin-2 (*LCN2*), the gene encoding NGAL, is transcriptionally promoted by NF-ƙB activation through toll-like receptors, IL-1β, or IL-17A/TNF (Cowland et al., 2006; Makhezer et al., 2019; Østvik et al., 2013a). Although NF-ƙB shows pleiotropic properties, most anti-inflammatory molecules decrease its activity, including CA and SFN (Treasure et al., 2023; Yang et al., 2017). Therefore, since NRF2 activation inhibits NF-ƙB activity, the NRF2 pathway is a logical target to assess potential protective effects of CA and SFN against inflammation.

Despite studies in chemical-induced colitis rodent models, CA or SFN have hardly been investigated in humans or human intestinal epithelial cells. The epithelium is the first part of the colonic tissue most drugs and diet compounds encounter after oral administration, so it is crucial to study its response to said molecules (Mansouri et al., 2025). Colonoids generated from patient tissue retain the genetic and disease specific characteristics of the parent tissue (Niklinska-Schirtz et al., 2021). Additionally, they respond to external stimuli and generate reproducible results in various molecular assays (Gopalakrishnan et al., 2023). Hence, the patient-derived colonoid model is a promising pre-clinical tool for drug discovery. In this study we determined the potential of colonoids as a platform to evaluate inflammation-related cellular responses induced by dietary phytochemicals, CA and SFN. We observed modulation of key inflammatory markers and activation of NRF2 target genes in colonoids as well as *LCN2*/NGAL modulation by both phytochemicals. Together, the data herein validates the use of human colonoids as a pharmacological testing model for phytochemicals and indicates the potential of CA and SFN to decrease inflammation in the epithelium.

## 2 Materials and methods

Materials are listed in Supplementary Table S1.

Raw data including absorbance, concentrations, quantification cycle (Cq) data, and densitometry are available at Mendeley Data (DO1: 10.17632/p8wkykmdnj.1).

### 2.1 Patient-derived colonoid culture and treatment

**FIGURE 2:**
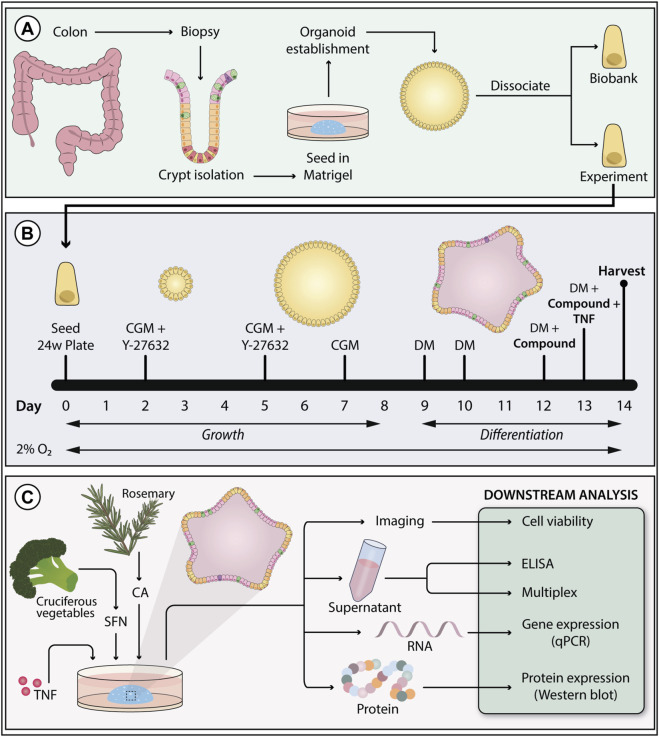
Colonoid establishment, experimental setup, and downstream analysis. **(A)** Colonoids were established from epithelial crypts isolated from patient colon biopsies. **(B)** Colonoids were grown in colonoid growth media (CGM) for 8 days to support stem cell renewal. The media was supplemented with the ROCK-inhibitor Y-27632 on day 0 and 2 to prevent anoikis. From day nine and throughout the experiments, colonoids were grown in differentiation media (DM) to promote epithelial maturation. Colonoids were exposed to treatments and tumor necrosis factor (TNF) stimulus in the last 3 days of differentiation. To better resemble *in vivo* colonic oxygen levels, colonoids were cultivated at 2% O_2_. **(C)** Colonoids treated with carnosic acid (CA) and sulforaphane (SFN) were harvested on day fourteen to perform the following downstream assays: cell viability, ELISA, chemokine 40-plex, qPCR (gene expression), and Western blot (protein expression).

All colonoids were taken from an internal biobank which was established from endoscopically uninflamed colonic mucosa of IBD and non-IBD patients admitted to St. Olav’s University Hospital for a colonoscopy (Figure 2A). The study was approved by the Central Norway Regional Committee for Medical and Health Research Ethics, Norway (reference numbers 22687 and 26789). All patients gave written informed consent; all the experimental methods were performed adhering to the principles of the Declaration of Helsinki. Variability across donors and experiments is greater for models containing multiple cell types. Thus, to assess the consistency of target cellular responses, several independent experiments on colonoids (passages 9–16) derived from three different donors were used in this work (1 non-IBD/2 UC, 1 female/2 males, ages 48/18/60, UC patients using 5-aminosalicylate and immunosuppressants).

The modified protocol followed to culture the colonoids was based on Mahe et al. (2015), Jung et al. (2011), said modifications have been previously described (Gopalakrishnan et al., 2023; 2022; Sæterstad et al., 2024). Composition and preparation of colonoid growth media (CGM) and differentiation media (DM) are described in Gopalakrishnan et al. (2023). The dietary phytochemicals tested in this work, SFN (2.5, 5.0, 10, 15, 20 μM, HY-13755, MedChem Express), and CA (12.5, 25, 50, 75, and 100 μM, PHR2208, Sigma-Aldrich), were dissolved in 0.02% or less of dimethyl sulfoxide (DMSO), which was used as vehicle control. Pro-inflammatory stimuli (TNF, 300-01A, PeproTech; IL-17A, 200–17, PeproTech) were dissolved in sterile water with 0.1% human serum albumin (A9731, Sigma-Aldrich). For colonoid expansion and experiments, single intestinal stem cells were resuspended in Matrigel® (356234, Corning, 7.3–8.1 mg/mL protein) at 15,000 cells/50 µL/well (expansion plates) or 9,000–10,000 cells/50 μL/well (experimental plates), seeded on pre-warmed tissue-culture treated 24-well plates (3524, Corning), and incubated for 20 min at 37 °C before adding 500 µL CGM. Expansion plates grew undifferentiated for 1 week at 5% O_2_. CGM was changed every other day, and Y-27632 (72304, STEMCELL Technologies) removed on day 5. Experimental plates were grown in a 2% O_2_ environment. On day 9, the differentiation process started by removing CGM and adding DM. Three days later, colonoids were pre-treated with the vehicle control DMSO, CA, or SFN in DM (Supplementary Figure S1). On day 13, DMSO, CA, or SFN treatments and pro-inflammatory stimuli (100 ng/mL TNF, 25 ng/mL IL-17A) were added in DM (Supplementary Figure S1) with half the concentration of A-83-01 (SML0788, Sigma-Aldrich). For the unstimulated controls, DMSO, CA, or SFN treatments without stimulus were added on day 13. The experimental timeline is presented in Figure 2B. After 24 h (Supplementary Figure S1), media and colonoids were collected for downstream analyses (Figure 2C).

### 2.2 Cell viability assay

Viability after treatments and pro-inflammatory stimulus of patient-derived colonoids was assessed with the ReadyProbes™ Cell Viability Imaging Kit (Blue/Red) (R37610, Invitrogen™). After media collection on day 14, new media was added to each well followed by one drop of the DAPI stain and one drop of the propidium iodide stain. After 10 min incubation at room temperature, pictures were taken with an Evos FL Auto 2 imager (Applied Biosystems) using the tgBFP channel (DAPI) and RFP channel (propidium iodide). Each treatment group had three technical replicates. Colonoids were imaged at 4x to capture the entire Matrigel® dome.

### 2.3 Human inflammatory chemokine panel, 40-plex

The Bio-Plex Pro™ Human Chemokine Panel kit (171AK99MR2, Bio-Rad) and a Bio-Plex 200 System (Bio-Rad) was used to screen forty inflammation modulating cytokines following the directions provided by the manufacturer; 6Ckine/CCL21, BCA-1/CXCL13, CTACK/CCL27, ENA-78/CXCL5, Eotaxin/CCL11, Eotaxin-2/CCL24, Eotaxin-3/CCL26, Fractalkine/CX3CL1, GCP-2/CXCL6, GM-CSF, Gro-α/CXCL1, Gro-β/CXCL2, I-309/CCL1, IFN-ϒ, IL-1β, IL-2, IL-4, IL-6, IL-8/CXCL8, IL-10, IL-16, IP-10/CXCL10, I-TAC/CXCL11, MCP-1/CCL2, MCP-2/CCL8, MCP-3/CCL7, MCP-4/CCL13, MDC/CCL22, MIF, MIG/CXCL9, MIP-1α/CCL3, MIP-1δ/CCL15, MIP-3α/CCL20, MIP-3β/CCL19, MPIF-1/CCL23, SCYB16/CXCL16, SDF-1α+β/CXCL12, TARC/CCL17, TECK/CCL25, TNF. On day 14, conditioned colonoid media was collected from three wells per treatment group and stored at −20 °C for a maximum of 1 week before analysis, or at −80 °C for long time storage. One technical replicate of each sample was run. Conditioned media which had not been previously thawed [except for the unstimulated samples in one individual experiment (Donor 1)] was used to minimize the number of degraded proteins in the samples.

### 2.4 Enzyme-linked Immunosorbent assay

Modulation of highly secreted pro-inflammatory cytokines CXCL1, CXCL8, and CXCL11, and NGAL was studied with R&D Systems DuoSet® ELISA kits: DY275 (CXCL1), DY208 (CXCL8), DY672 (CXCL11), and DY1757 (Lipocalin-2/NGAL). Kit guidelines were used to probe the samples. Because these were highly secreted proteins, media samples up to the third freeze/thaw cycle were used. Two technical replicates of each treatment group were added to pre-coated Nunc Maxisorp flat-bottom 96-well plates (442404, ThermoFisher) at working concentrations of 1:100 (CXCL1), 1:150 (CXCL8), undiluted or 1:2 (CXCL11), and 1:200 (Lipocalin-2/NGAL). Assay development was performed with the 3,3′,5,5′tetramethylbenzidine (TMB) substrate set (421101, BioLegend®) and the reaction stopped with 2N sulfuric acid (231-639-5, VWR). Absorbance was read at 450 nm and 570 nm wavelengths with the iMark™ microplate absorbance reader (Bio-Rad). Protein concentration was calculated with the MPM 6 software (Bio-Rad) using the 5-parameter logistic regression model.

### 2.5 RNA isolation, cDNA synthesis, and quantitative PCR

qPCR technique was used to quantify the expression of NRF2 target genes *HMOX1* and *SOD2*, as well as *LCN2* in the patient-derived colonoids. After media collection, Matrigel® domes were washed with Dulbecco’s Phosphate Buffer Saline (DPBS) (D8537, Sigma-Aldrich). Following DPBS removal, colonoids were resuspended in lysis buffer by scraping and syringe shearing, placed in a microcentrifuge tube, then stored at −80 °C until RNA isolation. RNA was isolated with the RNeasy® Mini Kit (74106, Qiagen) as per the manufacturer’s instructions. RNA concentration and quality was measured with the DS-11+ Spectrometer (DeNovix®). Extracted RNA was reversed transcribed into cDNA using the High-Capacity RNA-to-cDNA^TM^ Kit (4387406, Applied Biosystems) or Maxima First Strand cDNA Synthesis Kit for RT-qPCR (K1641, Thermo Scientific™) on a GeneAmp® PCR System 2700 (Applied Biosystems) following the manufacturer’s protocol. Real-time qPCR reactions were set up per the manufacture’s guidelines with the PerfeCTa SYBR Green FastMix, ROX (95073-012, Quantabio) or PowerTrack™ SYBR Green Master Mix for qPCR (A46012, Applied Biosystems) in MicroAmp™ Fast Optical 96-well reaction plates (4346907, Applied Biosystems). One ng of cDNA and 300 nM of pre-validated forward and reverse primer mixes (249900, Qiagen) were used in each reaction (three technical replicates per sample). *ACTB* was used as reference gene and the untreated colonoid media sample as the reference group. qPCR reactions were run in a StepOnePlus Real-Time PCR System (Applied Biosystems) with the following cycling protocol: 1) denaturing for 30 s/95 °C, 2) [PCR cycling of 3 s/95 °C into 30 s/60 °C] x40, and 3) melt curve of 15 s/95 °C into 1 min/60 °C into 15 s/95 °C. Cycling data was analyzed and 2^−ΔΔCq^ (RQ) values calculated with the StepOne software v2.3 (Applied Biosystems).

### 2.6 Protein extraction and Western blot

Western blot analysis was used to quantify the expression of target proteins in the patient-derived colonoids. The protein extraction protocol was based on published work by Gopalakrishnan et al. (2023), Gopalakrishnan et al. (2022). After media collection, Matrigel® domes were resuspended in Cell Recovery Solution (354253, Corning) and shook for 1 h on ice. The domes were centrifuged at 500 *g* and 4 °C for 5 min and the pellet collected, cleaned (first wash with Cell Recovery Solution followed by 3 DPBS washes), and stored at −80 °C until extraction. Pellets were lysed at 4 °C for 2 h while shaking in 30 µL lysis buffer [150 mM NaCl, 5 mM EDTA, 50 mM Tris-HCl pH 7.5, 1 mM dithiothreitol (A3668, MilliporeSigma), 1% NP-40, 1X cOmplete® EDTA-free protease inhibitor cocktail (11836170001, Roche), 1X phosphatase inhibitor cocktail I and III (P2850 and P0044, respectively, Sigma-Aldrich)]. Afterwards, samples were centrifuged at 13,000 g (4 °C) for 20 min and the supernatant collected. Protein concentration was measured with the Pierce™ BCA protein assay kit (23227, ThermoFisher). Protein lysates were denatured in 1X NuPAGE™ lithium dodecyl sulfate sample buffer (NP0007, Invitrogen) supplemented with 50 mM dithiothreitol at 70 °C for 10 min. Denatured samples as well as the PageRuler™ Prestained Protein Ladder (26616, ThermoFisher) were loaded into NuPage™ 4%–12% Bis-Tris gels (NP0321BOX or WG1402BX10, Invitrogen) and the electrophoresis ran with 1X NuPAGE™ MOPS SDS running buffer (NP0001, Invitrogen) at 180 V for 1 h and 30 min. Gels were electroblotted into 0.2 μm nitrocellulose membranes (1704159, Bio-Rad) using the Trans-Blot® Turbo Transfer System (Bio-Rad). Blots were blocked with blocking buffer for fluorescent Western blotting (MB-070, Rockland Immunochemicals, Inc.) for 1 h at room temperature followed by overnight incubation with primary antibody (Supplementary Table S1) at 4 °C. The next day, blots were washed with 1X tris-buffered saline buffer supplemented with 0.1% Tween 20 (TBST). Afterwards, blots were incubated with the appropriate secondary antibody [Goat anti-Rabbit IgG (H&L) Secondary Antibody DyLight™ 800 or Goat anti-Mouse IgG (H&L) DyLight™ 680 Conjugated, SA535571 and 35518, respectively, Invitrogen] for 1 h at room temperature followed by washes with 1X TBST and 1X tris-buffered saline. Finally, the blots were imaged with the Odyssey® Classic Imager (LI-COR) and the densitometry analysis of the bands performed with the Image Lab software (Bio-Rad).

### 2.7 Statistical analysis

Statistical analysis of the 40-plex cytokine assay was performed in RStudio (Version 4.4.2) with the lme4, and lmerTest packages (Bates et al., 2015; Kuznetsova et al., 2017). The R script used for the analysis is available at Mendeley Data (DO1: 10.17632/p8wkykmdnj.1). After removing samples with cytokine concentrations below 20 pg/mL and testing for homoscedasticity, only 24 cytokines/chemokines were viable for statistical analysis. Data was fitted to a linear mixed model and treatments were compared to their appropriate vehicle control using the Benjamini–Hochberg method for multiple testing, as previously described (Sridhar et al., 2025). Data from the ELISA, qPCR, and Western blot experiments were analyzed using the GraphPad Prism 10 software. ELISA and Western blot protein concentration data were converted to their Log2 values before statistical analysis to decrease interexperimental variation. All data sets were normally distributed, so treatments were compared to their appropriate vehicle control using repeated measures one-way ANOVA followed by Dunnett multiple comparisons test. All statistically different samples had a p-value of 0.05 or less and are indicated by * (p ≤ 0.05), ** (p ≤ 0.01), *** (p ≤ 0.001), or *** (p ≤ 0.0001).

## 3 Results

### 3.1 Carnosic acid and sulforaphane modulate cytokine secretion from TNF-stimulated patient-derived colonoids

**FIGURE 3:**
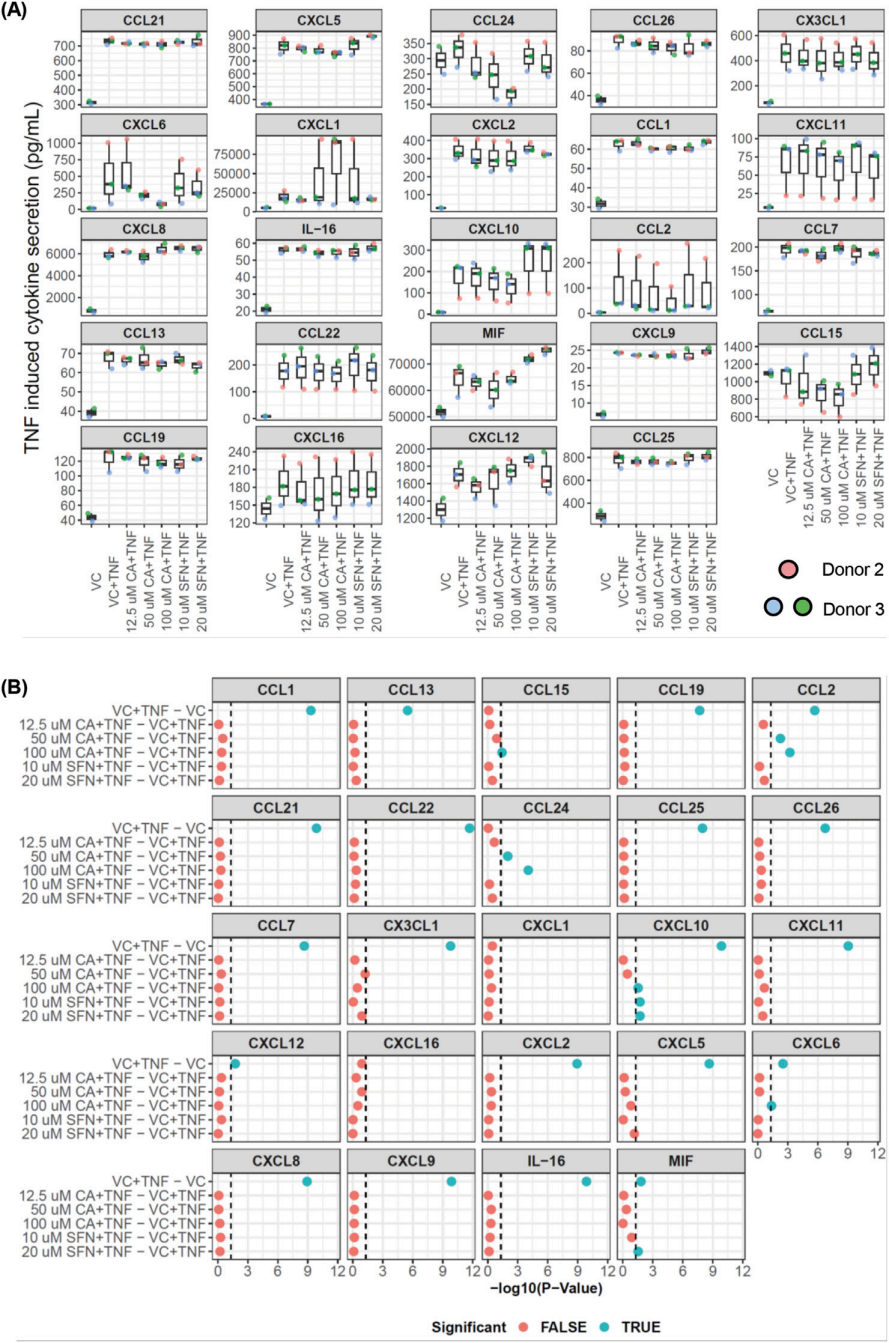
Detection of cytokines by 40-plex analysis of conditioned medium from tumor necrosis factor (TNF) stimulated patient-derived colonoid. As described in the method section, only 24 cytokines/chemokines were viable for statistical analysis after removing samples with cytokine concentrations below 20 pg/mL and testing for homoscedasticity. **(A)** The plots show the cytokine expression levels (pg/mL) across the different conditions: TNF-stimulated (100 ng/mL) colonoids pre-treated with vehicle control (VC, 0.02% DMSO), carnosic acid (CA, 12.5 µM, 50 μM, 100 µM), or sulforaphane (SFN, 10 μM, 20 µM). Data from *N* = 3 independent experiments with colonoids derived from Donor 2 and 3, as indicated by colored dots. **(B)** Panels show results from the linear mixed model used to evaluate significance between VC + TNF and the defined contrasts. Each panel represents a distinct cytokine, with the Y-axis indicating different comparisons and the X-axis representing the negative Log_10_ of adjusted p-values. The vertical dashed line at -Log10 (0.05) represents the threshold for statistical significance, and points to the right of these lines are considered statistically significant (blue dots). P values were adjusted using the Benjamini–Hochberg method for multiple testing.

Colonoid cell viability after CA or SFN treatment was tested with fluorescent stains added to the cultures (Supplementary Figure S2). Due to the 3D nature of the Matrigel® domes, the assay is not very sensitive, nevertheless, based on the acquired images, neither CA nor SFN at their highest concentrations appeared to kill the cells. As observed in the images presented in Supplementary Figure S1, TNF (100 ng/mL) induced some cell death that was not regulated by neither of the phytochemicals. Having established that the compounds were not toxic in our model system, broad anti-inflammatory effects of CA or SFN pre-treatment in patient-derived colonoids were explored with a 40-plex assay that tested for human cytokines, including chemokines, involved in inflammatory responses. The linear mixed model was employed to separately analyze effects of the compounds in the unstimulated (Supplementary Figure S3) and TNF-stimulated (Figure 3) conditions, compared to their appropriate vehicle controls (VC) (DMSO) or VC + TNF, respectively (treatments are listed in Supplementary Table S1).

CA and SFN treatments significantly modified the secretion of pro-inflammatory cytokines CCL20, CCL24, CXCL1, CXCL2, and macrophage migration inhibitory factor (MIF) in unstimulated colonoids (Supplementary Figures S3A, S3B). VC + TNF stimulation significantly induced secretion of 20 pro-inflammatory cytokines CCL21, CXCL5, CCL26, CX3CL1, CXCL6, CXCL2, CCL1, CXCL11, CXCL8, IL-16, CXCL10, CCL2, CCL7, CCL13, CCL22, MIF, CXCL9, CCL19, CXCL12, and CCL25 compared to VC (Figures 3A,B). When comparing CA and SFN pre-treated TNF-stimulated colonoids against VC + TNF stimulation, we found that pre-treatments with the compounds could regulate TNF induced secretion of CCL2, CCL15, CCL24, CXCL6, CXCL10, and MIF (Figures 3A,B). Both 50 μM and 100 µM CA statistically decreased CCL2, and CCL24 secretion in TNF-stimulated colonoids, while CCL15, CXCL6, and CXCL10 were only decreased by 100 µM CA. Interestingly, 100 µM CA also significantly decreased secretion of CCL24, CXCL1, and CXCL2 in unstimulated colonoids (Supplementary Figures S3A, S3B). Hence, the data suggests that modulation of CCL2, CCL15, CXCL6, and CXCL10 secretion by CA occurs through its anti-inflammatory activity. Meanwhile, the effect of CA on CCL24 secretion was independent of inflammatory stimuli and might indicate modulation of pathways unrelated to inflammation.

Both 10 μM and 20 µM SFN statistically increased secretion of CXCL10 and MIF in TNF-stimulated colonoids (Figures 3A,B); while decreasing CCL20, CXCL2, and increasing MIF in unstimulated colonoids (Supplementary Figures S3A, S3B). Together, the 40-plex screen showed that both CA and SFN treatment could slightly modulate secretion of some cytokines in unstimulated patient-derived colonoids. Pre-treatment with CA and SFN exhibited some opposing effects on TNF-induced cytokine secretion, with CA appearing to being more protective than SFN.

**FIGURE 4:**
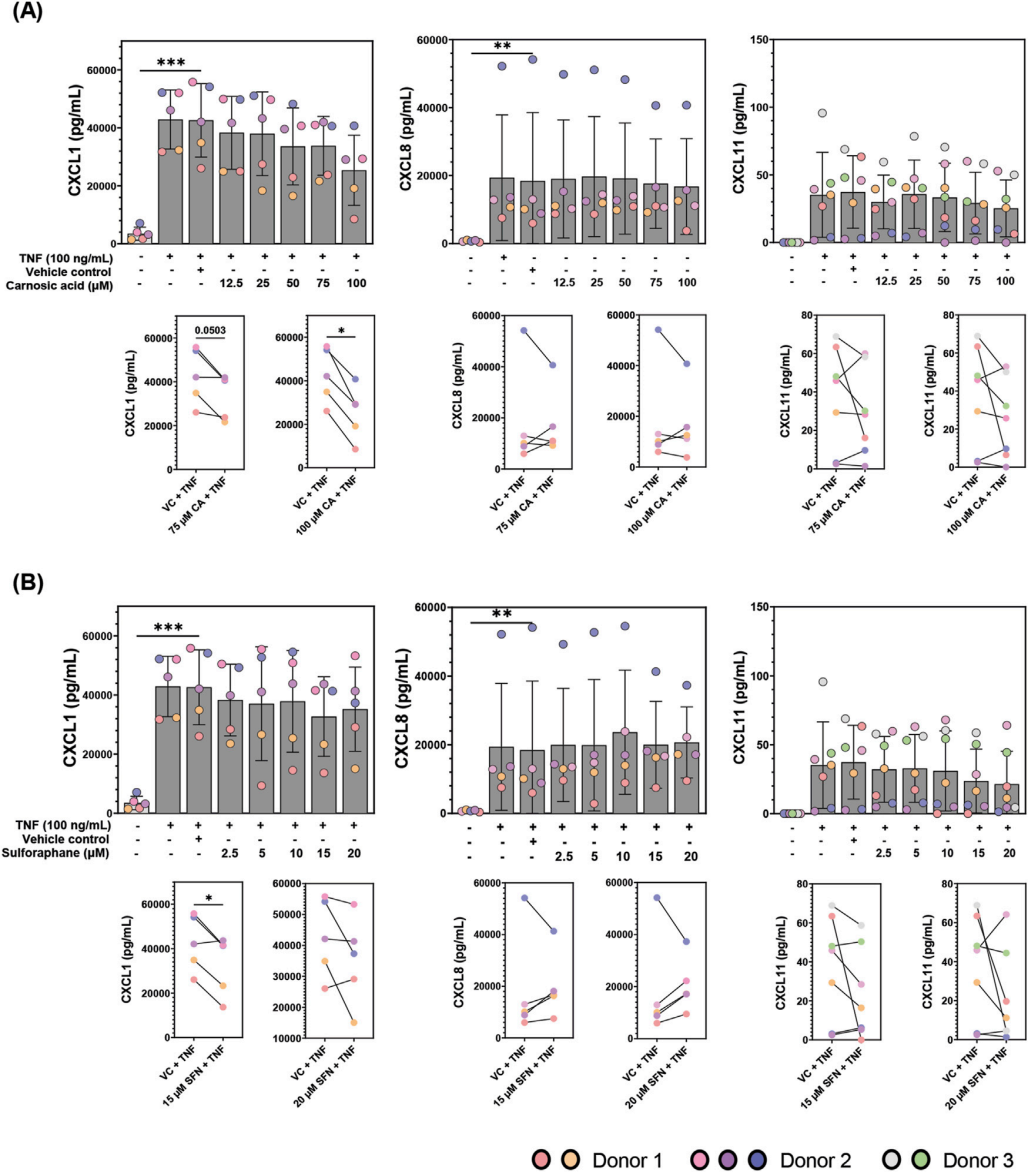
CXCL1, CXCL8, and CXCL11 concentrations (pg/mL) in the conditioned media of patient-derived colonoids pre-treated with **(A)** carnosic acid (CA, 12.5-100 uM), **(B)** sulforaphane (SFN, 2.5-20 uM), or vehicle control (VC, 0.02% DMSO) on day 12, followed by tumor necrosis factor (TNF, 100 ng/mL) stimulation on day 13. Data from *N* = 5-7 independent experiments with colonoids derived from Donor 1, 2, and 3, as indicated by colored dots. The cytokines were detected by ELISA and the Log2 transformed concentrations (pg/mL) were compared to the VC using repeated measures one-way ANOVA followed by Dunnett multiple comparisons test. CXCL1, CXCL8 CXCL11 levels (pg/mL) in TNF stimulated cultures pre-treated with the two highest concentrations of CA (75 and 100 µM) and SFN (15 and 20 µM) were compared to the VC + TNF using a paired t-test (paired plots). Total concentrations (pg/mL) were graphed for easier visualization. *p < 0.05, **p < 0.01, ***p < 0.001.

The 40-plex screen covered a broad range of cytokines; however, those near the upper detection limit such as CXCL1 may not be reliably measured. Previous characterization of patient-derived colonoids cultured at 2% O_2_ showed that they secrete pro-inflammatory chemokines CXCL1, CXCL8, and CXCL11 in high concentrations (Skovdahl et al., 2021). Hence, we employed specific ELISA assays to test these three highly secreted pro-inflammatory chemokines, aiming to further validate the inflammatory modulation by CA and SFN. There was no significant difference in the secretion of either of the chemokines after treatment of unstimulated colonoids with various doses of CA (12.5, 25, 50, 75, and 100 µM) or SFN (2.5, 5, 10, and 20 µM) (Supplementary Figure S5). As expected, TNF stimulation increased secretion of all three chemokines when compared to the untreated control (Figure 4). Pre-treatment with 75 and 100 µM CA (Figure 4A) or 15 µM SFN (Figure 4B) significantly attenuated TNF induced CXCL1 (Figures 4A,B). For CA, the strongest protective effects were observed with the highest dose (100 µM), as was also observed in the 40-plex. In most experiments, the compounds did not protect against TNF induced CXCL8 secretion, but pre-treatments with high doses of CA or SFN tended to attenuate TNF induced CXCL11. Overall, these results validate the use of TNF as pro-inflammatory stimulus in the patient-derived colonoids and showed that pre-treatment with both CA and SFN can decrease highly expressed inflammatory markers in said model. Therefore, this is a suitable platform to further test the anti-inflammatory properties of these two phytochemicals.

### 3.2 Pre-treatment with carnosic acid and sulforaphane upregulate transcription of the two NRF2 target genes, *HMOX1* and *SOD2* in TNF-stimulated patient-derived colonoids

**FIGURE 5:**
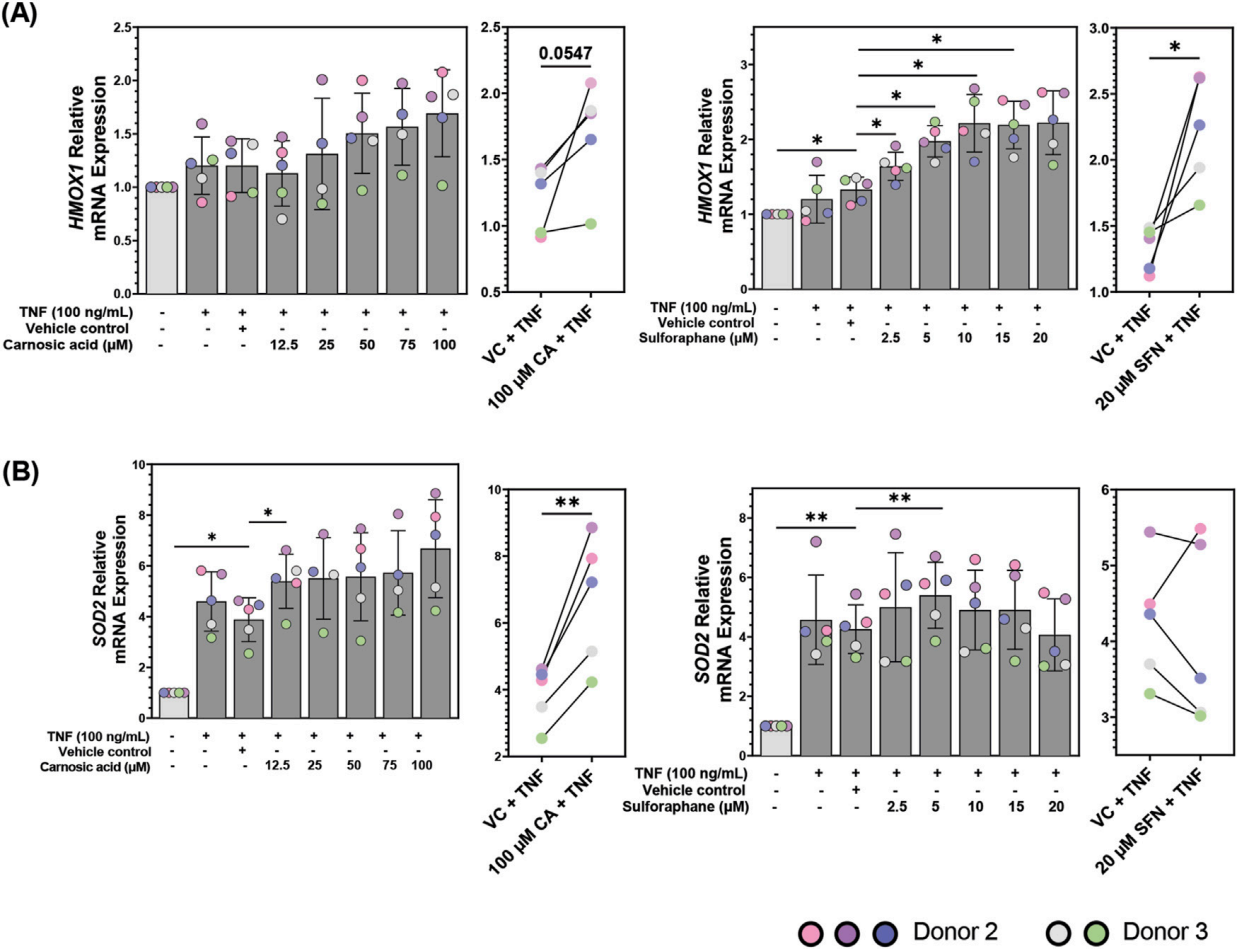
mRNA expression of **(A)** heme oxygenase 1 (*HMOX1)* and **(B)** superoxide dismutase 2 (*SOD2*) in patient-derived colonoids pre-treated with vehicle control (VC, 0.02% DMSO), carnosic acid (CA, 12.5–100 µM), or sulforaphane (SFN, 2.5–20 µM) on day 12, followed by tumor necrosis factor (TNF, 100 ng/mL) stimulation on day 13. Relative quantification (RQ = 2^−ΔΔCq^) values were normalized to the untreated media control. Data from *N* = 5 independent experiments with colonoids derived from Donor 2 and 3, as indicated by colored dots. Normalized RQ values were compared to the VC using repeated measures one-way ANOVA followed by Dunnett multiple comparisons test. The two highest concentrations of CA (75 and 100 µM) and SFN (15 and 20 µM) were compared to the VC using a paired t-test. *p < 0.05, **p < 0.01.

Both CA and SFN activate the NRF2 transcription factor (Hu et al., 2011; Satoh et al., 2008), thus modulation of NRF2 activity can be used to examine the bioactivity of both compounds. After validating that the TNF-stimulated patient-derived colonoids produced an inflammatory response and that CA and SFN could modulate cytokine secretion, we tested their promotion of NRF2 activity by measuring the transcriptional expression of two of its target genes, *HMOX1* and *SOD2* with the qPCR technique (Figure 5). *HMOX1* encodes the enzyme heme oxygenase 1 (HO-1) which degrades heme into carbon monoxide, bilirubin, and free iron (Campbell et al., 2021). Meanwhile, *SOD2* encodes the enzyme superoxide dismutase 2 (SOD2) which is confined to the mitochondria and tasked with converting superoxide into hydrogen peroxide at the end of the electron transport chain (Palma et al., 2020). These enzymes feature the two types of proteins that are transcribed by NRF2, detoxifying and antioxidant, respectively.

TNF did not significantly upregulate *HMOX1* mRNA expression in the colonoids (Figure 5A). CA and SFN alone induced less than 0.5-fold increase compared to unstimulated control (Supplementary Figure S6). Pre-treatment with high dose of CA (100 µM) tended to upregulate *HMOX1* compared to VC + TNF (p = 0.0547). SFN pre-treatments dose-dependently and significantly upregulated *HMOX1* mRNA expression in TNF-stimulated colonoids from 2.5 µM plateauing at 10 µM (Figure 5A, right panel). Thus, during TNF-induced inflammation in colonic epithelial cells, CA and particularly SFN may offer protection by regulating cellular stress response. In contrast to *HMOX1*, TNF itself induced a strong and significant upregulation of *SOD2* mRNA compared to unstimulated control. Pre-treatment with 100 µM CA further enhanced the SOD2 expression (Figure 5B, left panels). Pre-treatment with 5 µM SFN induced a small, but significant increase in SOD2 mRNA compared to VC + TNF. Further, SFN showed a biphasic effect downregulating *SOD2* transcription in four out of five experiments at 20 µM. (Figure 5B, right panels). Neither CA nor SFN promoted transcription of *SOD2* in unstimulated colonoids (Supplementary Figure S6B). Therefore, inflammatory stimulus which generates oxidative stress is needed for SOD2 transcription, making it a good marker of inflammation in these colonoids as well as a selective target for NRF2 activity in the inflamed colonoids.

### 3.3 NRF2, p65, HO-1 and SOD2 proteins are expressed by patient-derived colonoids 24 h after TNF stimulation

**FIGURE 6:**
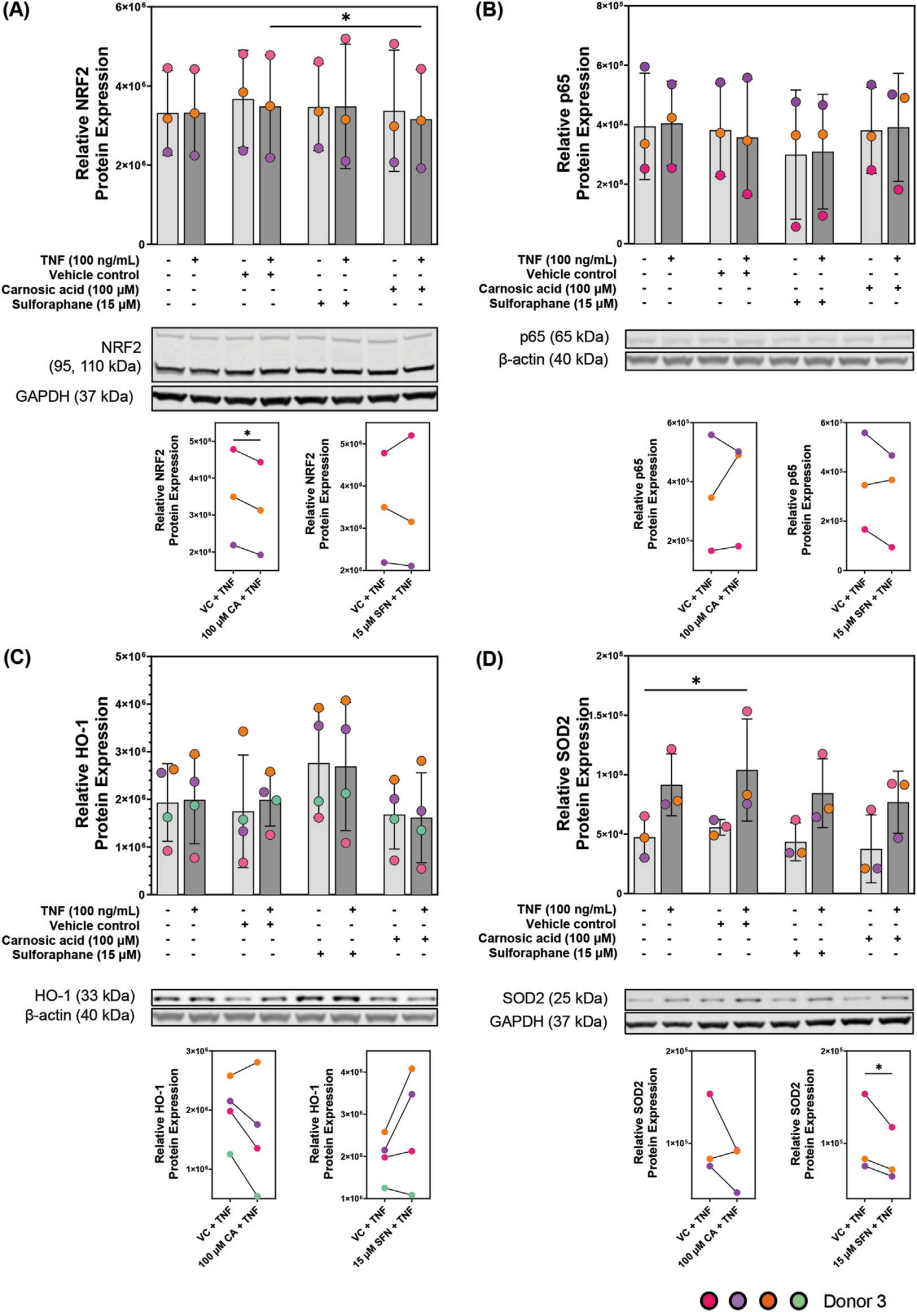
Protein expression of **(A)** nuclear factor erythroid 2-related factor 2 (NRF2), **(B)** p65, and NRF2 transcription targets **(C)** heme oxygenase 1 (HO-1) and **(D)** superoxide dismutase 2 (SOD2) in patient-derived colonoids. Unstimulated and 100 ng/mL tumor necrosis factor (TNF) stimulated colonoids were pre-treated with vehicle control (VC, 0.02% DMSO), 100 µM carnosic acid (CA), or 15 µM sulforaphane (SFN). Blots shown are representative images of *N* = 3 or 4 independent experiments (colored dots) with colonoids derived from Donor 3. Log2 transformed absolute values were compared to the VC using repeated measures one-way ANOVA followed by Dunnett multiple comparisons test. Total absolute densities were graphed for easier visualization. *p < 0.05. Full blots are available in Supplementary Figure S7.

In the cytokine secretion and mRNA expression analysis (*HMOX1* and *SOD2*), the concentrations of 100 µM CA and 15 µM SFN were found most effective. Therefore, these concentrations were selected for protein expression analysis of NRF2, the p65 subunit of NF-ƙB, HO-1, and SOD2 using Western blot technique (Figures 6A–D, respectively). All four proteins were expressed in the colonoids. Untreated unstimulated and TNF-stimulated colonoids showed no difference in NRF2 protein expression compared to untreated control or VC, and SFN pre-treatment also did not change the expression. Pre-treatment with 100 µM CA induced a small (∼0.02-fold change) but statistically significant downregulation of NRF2 protein expression in the TNF-stimulated colonoids (Figure 6A). NF-ƙB is an important and well-studied transcription factor that promotes transcription of genes encoding pro-inflammatory proteins (Hayden and Ghosh, 2008). Additionally, NRF2 activation negatively represses NF-ƙB activity (Saha et al., 2020). There are five subunits that can dimerize into NF-ƙB; p65, p50, p52, c-Rel, and RelB (Hayden and Ghosh, 2008). As p65 is the most abundant subunit with transcriptional activity, we measured its expression as representation of NF-ƙB in the colonoids (Figure 6B). TNF-stimulated colonoids showed no p65 upregulation, and pre-treatment with CA or SFN did not influence its protein expression. NRF2 and NF-ƙB are transcription factors, thus their activation and protein upregulation occur soon after inflammatory stimulus. Hence, their protein concentrations usually peak between 1–6 h after stimulus and by 24 h their expression has decreased to basal levels (Hoffmann and Baltimore, 2006; Khor et al., 2006; Ma, 2013; Rogler et al., 1998). This phenomenon most likely explains the lack of differential expression by CA and SFN on NRF2 and p65 protein expression.

HO-1 and SOD2 are targets of a transcription factor, thus, their peak protein expression should be after the peak of the transcription factor. However, at 24 h, we observed a tendency to decrease protein expression compared to the VC; except in SFN for HO-1. There was no observed difference between unstimulated and TNF-stimulated colonoids after VC, CA, nor SFN pre-treatment (Figures 6C,D). Nevertheless, 15 µM SFN clearly increased HO-1 protein expression following the trend observed at the mRNA level. Meanwhile, 100 µM CA weakly decreased HO-1 protein expression when compared to the VC. While TNF stimulus upregulated SOD2 protein expression, both CA and SFN pre-treatments weakly decreased its expression when compared to the VC. As with the transcription factors, 24 h seems to be the wrong time to measure changes in HO-1 and SOD2 protein expression in this model. These two enzymes have been known to have peak expression between 6–24 h (Murley et al., 2007). Thus, time-dependent experiments are needed to better assess the modulation of these proteins by CA and SFN in the patient-derived colonoids.

### 3.4 Carnosic acid and sulforaphane decrease neutrophil gelatinase-associated lipocalin protein expression and secretion in TNF-stimulated patient-derived colonoids

**FIGURE 7:**
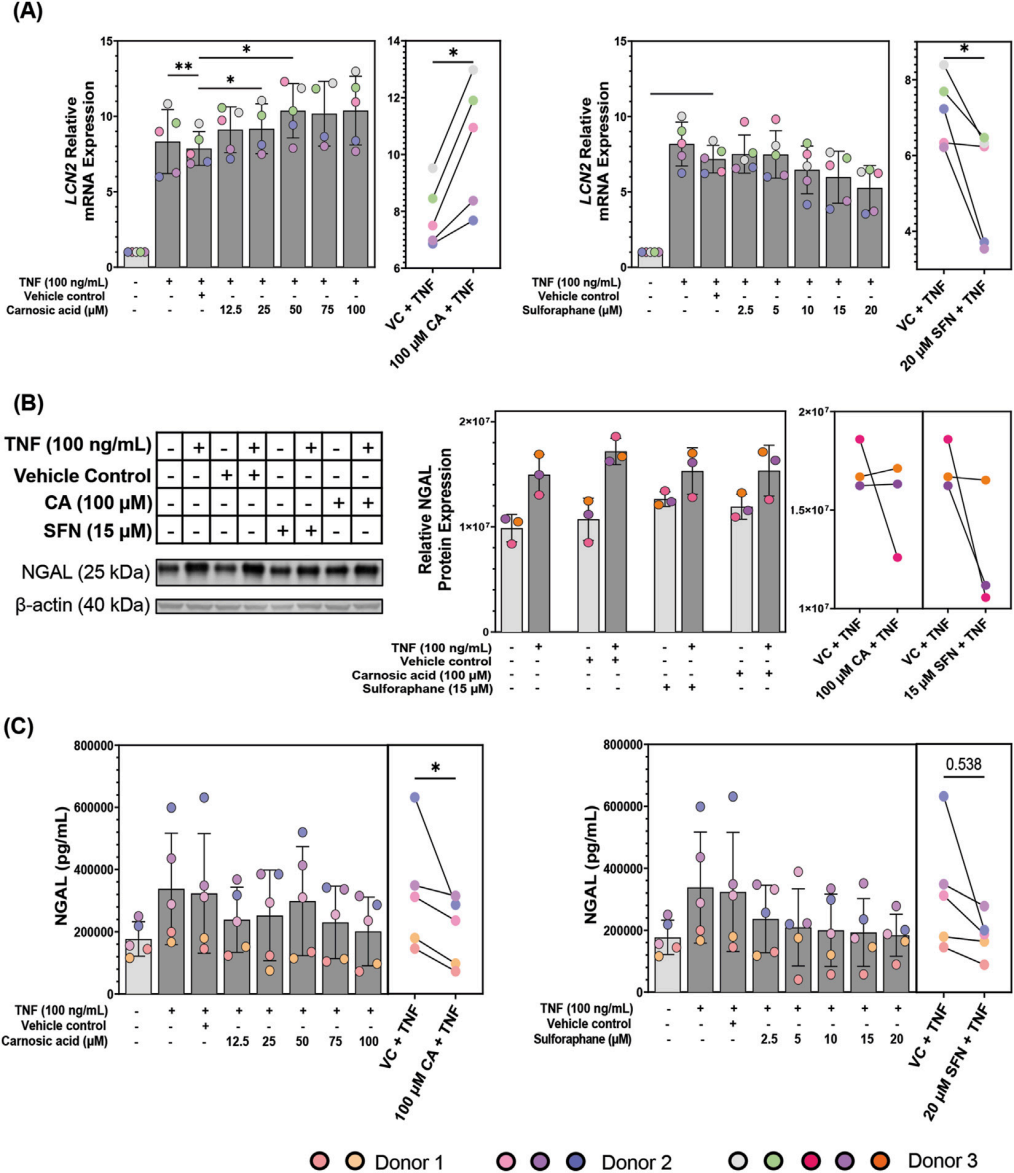
Neutrophil gelatinase-associated lipocalin (NGAL)/lipocalin-2 (*LCN2*) modulation in patient-derived colonoids. **(A)** *LCN2* mRNA expression in tumor necrosis factor (TNF, 100 ng/mL) stimulated colonoids pre-treated with vehicle control (VC, 0.02% DMSO), carnosic acid (CA, 12.5–100 µM), or sulforaphane (SFN, 2.5–20 µM). Relative quantification (RQ = 2^−ΔΔCq^) values were normalized to that of the untreated media control. Data from *N* = 5 independent experiments with colonoids from Donor 2 and 3, as indicated by colored dots. **(B)** NGAL protein expression in unstimulated and TNF-stimulated (100 ng/mL) colonoids pre-treated with VC, 100 μM CA, or 15 µM SFN. Blots shown are representative images of *N* = 3 independent experiments (colored dots) with colonoids from Donor 3. **(C)** NGAL secretion levels in the media of TNF (100 ng/mL) stimulated colonoids pre-treated with vehicle control (VC, 0.02% DMSO), carnosic acid (CA, 12.5–100 µM), or sulforaphane (SFN, 2.5–20 µM). Data from *N* = 5 independent experiments with colonoids derived from Donor 1 and 2, as indicated by colored dots. Secreted NGAL concentrations (pg/mL) were transformed into Log2. Normalized RQ values, Log2(NGAL concentrations), and Log2 (absolute densities) were compared to the VC using repeated measures one-way ANOVA followed by Dunnett multiple comparisons test. The highest concentration of CA and SFN were compared to the VC using a paired t-test for **(A)** and **(C)**. Total NGAL concentrations (pg/mL) and total absolute densities were graphed for easier visualization. *p < 0.05, **p < 0.01. Full blots are available in Supplementary Figure S8.

Pharmacologically, NGAL is involved in epithelial remodeling and regeneration (Schmidt-Ott et al., 2007; Yan et al., 2001), and its expression is inflammation-dependent, thus it could also be a good pharmacological inflammation marker in epithelial cells. Hence, we studied the effects of CA and SFN pre-treatment on *LCN2* (gene that encodes NGAL) mRNA expression. TNF induced an ∼8-fold change in *LCN2* mRNA expression compared to unstimulated controls (Supplementary Figure S7A), validating strong *LCN2* expression in inflamed epithelial cells. While SFN statistically downregulated *LCN2* mRNA expression in TNF-stimulated colonoids when compared to the VC, CA statistically upregulated it (Figure 7A). Both effects were dose dependent. In unstimulated colonoids, higher concentrations of CA increased *LCN2* expression (0.3- to 0.5-fold) and SFN decreased it (∼0.2-fold) (Supplementary Figure S7A), following the trends observed in stimulated colonoids. Thus, the effects of CA and SFN are partly independent on inflammatory stimulus, and the observed small fold-changes (0.2–0.5) might correlate with the low transcription of *LCN2* in uninflamed cells.

After confirming that CA and SFN influence *LCN2* mRNA expression we looked at the effect of 100 µM CA and 15 µM SFN on NGAL protein expression in the colonoids (Figure 7B). As expected, NGAL expression was higher in TNF-stimulated colonoids. CA tended towards upregulation of NGAL protein expression in unstimulated colonoids, aligning with the results from the mRNA expression. Interestingly, SFN also tended towards NGAL protein expression upregulation in the unstimulated colonoids. CA nor SFN pre-treatment significantly changed NGAL protein expression in the TNF-stimulated colonoids when compared to the VC + TNF but trended towards downregulation.

Pharmacological agents could also modulate NGAL’s secretion, so we measured the secretion of NGAL in the patient-derived colonoids with an ELISA assay. NGAL secretion was minimally modulated by CA or SFN treatment in unstimulated colonoids, CA tending towards upregulation (Supplementary Figure S7B). Continuing the trend of the mRNA and protein expression assays, TNF-stimulated colonoids secreted more NGAL than unstimulated colonoids (Figure 7C). In TNF-stimulated colonoids, CA and SFN pre-treatments dose-dependently decreased NGAL protein secretion (Figure 7C). The only concentrations that did not have significant difference were 50 µM CA and 20 µM SFN.

The incongruence between modulation of *LCN2* mRNA expression and NGAL protein expression and secretion by CA was unexpected, even more so because SFN showed congruency between assays. Both mRNA and protein expression and secretion were analyzed 24 h after TNF stimulus, thus the data are not contingent on each other. The gene expression pathway states that translation occurs after transcription, so the mRNA produced at 24 h will be reflected at the protein level following those 24 h (Buccitelli and Selbach, 2020). Also, secretion happens after translation further elongating the timeline. Therefore, the obtained data indicates the effect of CA and SFN on the state of *LCN2* transcription and NGAL protein expression and secretion 24 h after TNF stimulus. One interesting observation was the much stronger TNF-stimulated increase at the mRNA level compared to the protein level, and even further at the level of secretion. This could be due to differences in regulation of these processes and degradation rates (Buccitelli and Selbach, 2020). Nevertheless, CA decreased levels in both protein assays, thus its upregulating effects are only at the mRNA level. NGAL influences the cells after its secretion, thus lowering secretion is the main goal when it comes to this protein; and both CA and SFN do so effectively.

## 4 Discussion

Use of 3D organoid culture is at the forefront of *in vitro* pharmacological testing because they recapitulate the physical and molecular structure of their parent tissue (Zhao et al., 2022). Once established, organoids can be used in an “immortalized cell line manner” and passaged without genetical changes, allowing for reproducible results. Organoids derived from patient tissue are most physiologically relevant, since disease traits do not have to be genetically or chemically induced, and patient variability can be studied and considered. In this study we utilized epithelial organoids derived from uninflamed colonic biopsies of UC patients and non-IBD controls, since organoids from inflamed tissue are extremely difficult to establish and anyway restore an uninflamed profile upon *in vitro* culturing (Arnauts et al., 2020; Hammerhøj et al., 2025). Previous work by Walaas et al., showed that patient-derived colonoids retain the same phenotype when cultured at 20% (standard cell culturing level) or 2% O_2_ (physiological hypoxia/physioxia) (Walaas et al., 2023). However, culturing at 2% O_2_ altered their transcriptome, improving growth and functional differentiation. Therefore, we cultured colonoids at 2% O_2_ to further increase the physiological relevance of the model. As with any model, colonoids have limitations: 1) we can only study effects in the epithelium and involvement of immune cells requires co-culturing, 2) the lumen is inside the organoid structure, so the phytochemicals only interact with the basal part of the structure which will not account for apical absorption, 3) not all stem cells isolated from patient tissue develop into colonoids, and 4) it is costly. Nevertheless, colonoids are an underutilized pharmacological platform which, in the future, could be used for personalized drug screening (Wang et al., 2022).

In this study we tested the anti-inflammatory potential of the dietary phytochemicals CA and SFN, both found in high concentrations in their respective plants, socially valued for their health promoting effects, consumed in high amounts, and have proven anti-inflammatory capability (Birtić et al., 2015; Treasure et al., 2023). Therefore, they were good candidates for examining the viability of patient-derived colonoids as testing model for phytochemicals which may have more promiscuous effects and could generate more metabolites compared to synthetic drugs. To mimic inflammation, colonoids were stimulated with 100 ng/mL TNF for 24 h after a 24 h pre-treatment with CA or SFN. In the data herein presented we compared the unstimulated and TNF-stimulated samples only to their corresponding vehicle control because we were interested in treatment efficacy. To assess whether TNF stimulus was sufficient to elicit an inflammatory response and whether CA and SFN pre-treatments were able to modulate it, we first employed a multiplex assay of 40 cytokines involved in inflammation. Only 24 cytokines were expressed above our set threshold of 20 pg/mL (Supplementary Figure S3, S4). CA and SFN significantly regulated CCL20, CCL24, CXCL1, CXCL2 and MIF in unstimulated colonoids (Supplementary Figure S3, S4). TNF stimulus significantly induced secretion of 20 cytokines, all known to be expressed by the colonic epithelium (Neurath, 2014; Wang et al., 2009). CA decreased secretion of CCL2, a monocyte and macrophage chemoattractant; CCL15, a monocyte and macrophage chemoattractant with anti-microbial properties; CCL24, an eosinophil, basophil, and Th2 chemoattractant; CXCL6, a neutrophil chemoattractant that upregulates MMP-9; and CXCL10, an activated T cell, specially Th1 cells, chemoattractant in TNF-stimulated colonoids. These chemokines are pro-inflammatory and overexpressed in patients with active UC (Chen et al., 2001; Gijsbers et al., 2004; Kotarsky et al., 2010; Østvik et al., 2013b; Singh et al., 2016a). Thus, CA statistically decreased inflammatory markers associated with UC in the TNF-stimulated patient-derived colonoids. CA’s modulation of CCL24 was particularly interesting because to our knowledge it has not been reported before. Moreover, the decrease in secretion was independent of pro-inflammatory stimulus, which might negate involvement of inflammatory pathways which are commonly associated with CA’s medicinal activity. In future studies, the immunoregulatory effects of CA in epithelial cells should be explored more in depth, since there is minimal published work describing the effect of CA on these chemokines.

MIF is a pleiotropic protein whose overexpression is associated with several intracellular functions such as induction of MMP-1 and MMP-9 expression, proliferation, and wound healing (De Jong et al., 2001; Farr et al., 2020; Nishihira, 2012). Some reported extracellular functions are upregulation of pro-inflammatory cytokine secretion, and neutrophil and macrophage chemoattraction (Nishihira, 2012). Although the literature shows that SFN binds and inhibits MIF (Treasure et al., 2023), SFN differentially increased MIF secretion in the patient-derived colonoids. The observed upregulation might be a transient compensatory mechanism employed by the cells to account for inactivated MIF (Brown et al., 2009; Healy et al., 2011). This phenomenon has been observed with glutathione levels in different cell types as well as *in vivo* (Treasure et al., 2023). CA also showed nominal MIF increase without statistical significance. High MIF concentrations make the 40-plex a poor assay to study its secretion levels because of the decreased sensitivity at the upper detection limit. MIF is expressed by intestinal epithelial cells, and positively correlated with IBD risk (Song et al., 2024; Yang et al., 2015). However, expression of known MIF receptor CD74 is increased in colonic tissue from IBD patients and its elimination showed UC symptom worsening (Farr et al., 2020). Therefore, positive modulation of MIF by SFN should be further investigated to better understand whether there are any ramifications to MIF overexpression and if they are beneficial or detrimental.

The 40-plex assay was used as a screening method because of the inherent bias it has against values near the detection limits. Thus, we validated the results by measuring the secretion of three pro-inflammatory chemokines which are highly expressed by the patient-derived colonoids after inflammatory stimulus (Skovdahl et al., 2021). These chemokines were CXCL1 which recruits neutrophils and is transcribed by NF-ƙB in UC patient tissue and DSS colitis mice model (Xu et al., 2022); CXCL8 which recruits neutrophils and granulocytes, promotes tumor growth and cell migration, and modulates inflammation (Zhu et al., 2021); and CXCL11 which is involved in IL-6 mediated inflammation as well as Th17 cell development by IL-6 upregulation (Liu et al., 2011). Both CA and SFN seemed to downregulate TNF-stimulated secretion of the three chemokines, yet only CXCL1 reached statistical significance (Figure 4). The regulation of CXCL8 was most variable (Figure 4), possibly due to the multiple transcription factors and regulation mechanisms that can promote CXCL8 in UC (Zhu et al., 2021). The attenuating effect of CA and SFN on secreted pro-inflammatory chemokines was only seen in the TNF-stimulated colonoids, indicating that their effect is inflammation dependent. In conjunction with the 40-plex data, these results show that the patient-derived colonoids are a good model to test the anti-inflammatory potential of phytochemicals like CA and SFN.

The NRF2 transcription factor is part of an endogenous antioxidant pathway that modulates inflammation (Ma, 2013) and is expressed in unstimulated and TNF-stimulated patient-derived colonoids as observed in the protein expression assay (Figure 6A). Electrophilic molecules like CA and SFN activate the pathway by binding to its cytoplasmic repressor kelch-like ECH-associated protein 1 (Keap1) (Dinkova-Kostova et al., 2017; Satoh et al., 2008). These two characteristics made this a good pathway to investigate modulation by CA and SFN, as well as the use of colonoids to study mechanisms of action. CA and SFN pre-treatments dose-dependently upregulated mRNA expression of two NRF2 target genes, *HMOX1* and *SOD2*, with statistical significance in TNF-stimulated colonoids (Figure 5). Therefore, both phytochemicals activate NRF2 in this model suggesting that CA and SFN are targeting the NRF2 pathway to decrease inflammation in the colonoids. The ROS SOD2 and other antioxidant proteins neutralize, activate the NF-ƙB which promotes transcription of pro-inflammatory cytokines like CXCL1 and CXCL8 (Morgan and Liu, 2011; Xu et al., 2022; Zhu et al., 2021). Also, NRF2 itself inhibits the degradation of the NF-ƙB repressor inhibitor of nuclear factor kappa-B kinase subunit beta (i.e., IKKβ), thus blocking NF- ƙB nuclear translocation (Peng et al., 2023).

SFN pre-treatment tended towards HO-1 protein expression upregulation, yet CA tended towards a slight decrease (Figure 6C). Unexpectedly, neither CA nor SFN had a strong effect on SOD2 protein expression (Figure 6D). This assay showed that both proteins are expressed in the patient-derived colonoids, but 24 h after TNF stimulation is not the ideal timepoint to observe changes in their expression. Although unexpected, it was not surprising that NRF2 protein expression returned to baseline after 24 h of TNF stimulus because transcription factor peak expression time is 1 h–6 h after induction. Thus, CA and SFN promoted NRF2 transcriptional activity, but it is unclear whether said changes translate into protein expression. In a future study we need to employ a time series to assess which is the best time to observe changes for each protein. Nevertheless, proteins of interest were expressed at quantifiable amounts in patient-derived colonoids, and TNF stimulus increased the expression of some of these proteins. Hence, this model is a good platform to study phytochemical modulation of mRNA and protein expression.

The NF-ƙB transcription factor plays an important role in inflammatory responses, UC included, and it is expressed in intestinal epithelial cells (Rogler et al., 1998; Schreiber et al., 1998). Besides being negatively regulated by NRF2, NF-ƙB promotes *LCN2* transcription in intestinal epithelial cells (Saha et al., 2020; Stallhofer et al., 2015). p65 is the most abundant transcriptionally active NF-ƙB subunit, thus we measured its expression in the patient-derived colonoids (Figure 6B). CA and SFN pre-treatments had no effect on p65 protein expression which could have been because of the timing after stimulation and should not be taken as conclusive data.

NGAL is a pleiotropic protein highly secreted by inflamed epithelial cells (Liu et al., 2019; Saha et al., 2017; Singh et al., 2016b; Thorsvik et al., 2019; Yan et al., 2001). Fecal NGAL concentrations are reported to positively correlate with active UC, and NGAL has thus been proposed as a biomarker for UC (Zollner et al., 2020). In a pharmacological setting, NGAL expression could also be a good inflammation marker in epithelial cells because of its involvement in epithelial remodeling and regeneration (Schmidt-Ott et al., 2007; Yan et al., 2001), and its high and inflammation-dependent expression. Hence, we studied the effects of CA and SFN pre-treatment on *LCN2* (gene that encodes NGAL) mRNA expression. Both CA and SFN downregulated NGAL secretion and intracellular expression, however, CA significantly upregulated *LCN2* mRNA while SFN downregulated *LCN2*. mRNA and protein expressions are not always correlated because many processes, like post-translational modifications, can alter the protein and not the mRNA (Buccitelli and Selbach, 2020). Also, CA might be modulating a protein that is not negatively regulated by NRF2 or is independent of it, and that is somehow involved in *LCN2* transcription. NGAL modulation by SFN and CA might be an indirect result of their effects on inflammation and not a direct tempering in the NGAL pathway. One of the reasons NGAL is overexpressed in the inflamed colon could be its role in wound healing (Thorsvik et al., 2019). Since cells in inflamed tissues die, the tissue needs to regenerate, thus it will have a higher number of progenitor cells. NGAL is highly expressed in undifferentiated cells, thus damaged areas during regeneration will produce more NGAL. Therefore, by decreasing inflammation you will have less damaged tissue in need of repair and lower levels of NGAL. However, this indirect effect would not explain the difference in *LCN2* mRNA regulation by CA or the decrease in secretion at peak protein expression. Hence, more work is needed to elucidate how CA and SFN modulate *LCN2*/NGAL in patient-derived colonoids.

**FIGURE 8:**
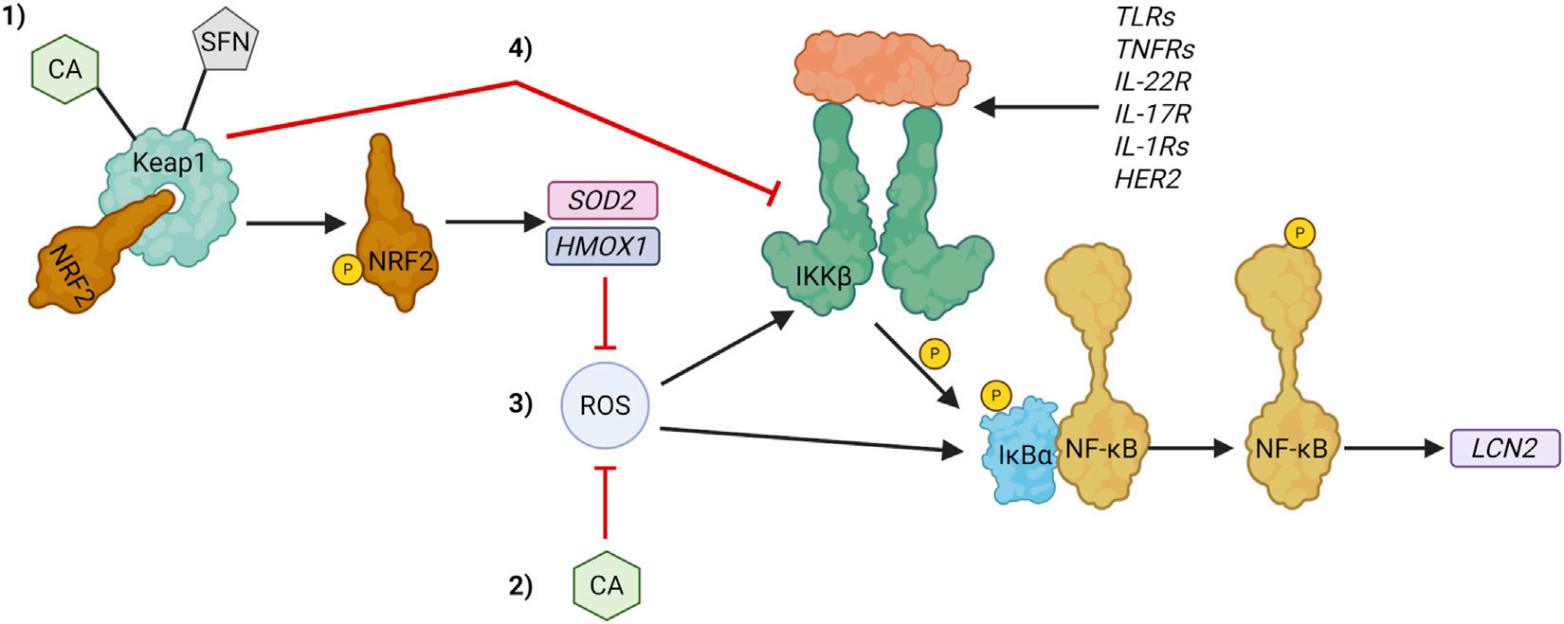
Possible mechanism of action for the antioxidant and anti-inflammatory activities of carnosic acid (CA) and sulforaphane (SFN) in tumor necrosis factor-stimulated patient-derived colonoids. 1) CA and SFN electrophilically bind cysteines in kelch-like ECH-associated protein 1 (Keap 1) promoting nuclear factor erythroid 2-related factor 2 (NRF2) detachment from Keap 1. Afterwards, free cytosolic NRF2 translocates to the nucleus where it promotes transcription of antioxidant and detoxifying target genes like superoxide dismutase (*SOD2*) and heme oxygenase-1 (*HMOX1*), respectively. 2) CA directly neutralizes reactive oxygen species (ROS) through oxidation-reduction reactions. Both pathways decrease the activity of the pro-inflammatory transcription factor nuclear factor kappa-light-chain-enhancer of activated B cells (NF-ƙB). 3) ROS increase NF-ƙB activity by promoting IKKβ and IƙBα phosphorylation. They can also directly bind to NF-ƙB to enhance DNA binding. 4) NRF2 inhibits the inhibitor of nuclear factor kappa-B kinase subunit beta (IKKβ) preventing phosphorylation of the NF-ƙB cytosolic repressor inhibitor of kappa-B alpha (IƙBα). IƙBα phosphorylation promotes NF-ƙB detachment from IƙBα leading to NF-ƙB nuclear translocation and subsequent promotion of target genes like lipocalin-2 (*LCN2*). Created in BioRender. Rivera (2025) https://BioRender.com/ec3npzw.

Together, the data presented in this study supports the use of patient-derived colonoids cultured at 2% O_2_ level as an *in vitro* testing platform for medicinally active phytochemicals. Like other models, patient-derived colonoids have multiple limitations (e.g., phytochemical basal absorption and lack of non-epithelial cells), still the model is a valuable tool that sheds light into pharmacological behavior in human tissue. Our data showed that CA pre-treatment decreased the secretion of pro-inflammatory cytokines CCL2, CCL15, CCL24, CXCL1, CXCL6, CXCL10, and CXCL11, while SFN pre-treatment decreased CXCL1 and CXCL11. Both phytochemicals increased expression of NRF2 target genes, and modulated *LCN2*/NGAL expression. Therefore, NRF2 activation might be the pathway CA and SFN target to decrease inflammation in this model (Figure 8). Additionally, CA might be directly neutralizing ROS (Figure 8). Increasing NRF2 activity usually decreases the activity of NF-ƙB which promotes the transcription of pro-inflammatory proteins like cytokines. *LCN2* expression data suggested that SFN decreased NF-ƙB transcriptional activity while CA increased it. Thus, additional studies are necessary to fully understand how CA and SFN modulate inflammation in patient-derived colonoids.

## Data Availability

The datasets presented in this study can be found in online repositories. The names of the repository/repositories and accession number(s) can be found below: https://data.mendeley.com/preview/p8wkykmdnj?a=bff19bba-756e-4938-8848-3618a9d285e4, 10.17632/p8wkykmdnj.1.
