# Supplementary material for: The dietary phytochemicals carnosic acid and sulforaphane regulate inflammatory markers in ulcerative colitis patient-derived colonoids
Source: Front Pharmacol. 2025 Dec 18;16:1696576. doi: 10.3389/fphar.2025.1696576 (PMC12756672; doi:10.3389/fphar.2025.1696576)
Supplement: Supplementary file 1 [file DataSheet1.pdf]

Supplementary Table 1.

Treatment	Purpose
Untreated	Blank control
100 ng/mL TNF	Inflammatory stimulus
0.02% DMSO	Vehicle control for unstimulated samples
0.02% DMSO + 100 ng/mL TNF	Vehicle control for TNF-stimulated samples

Name	Concentration	Catalog Number	Manufacturer
**Colonoid culture and treatment reagents**			
Carnosic acid (CA), 98.74% Purity	12.5, 25, 50, 75, 100 µM	HY-N0644	MedChem Express
Sulforaphane (SFN), 98.87% Purity	2.5, 5.0, 10, 15, 20 µM	HY-13755	MedChem Express
Recombinant human Tumor Necrosis Factor (TNF)	100 ng/mL	300-01A	PeproTech
Interleukin 17A (IL-17A)	25 ng/mL	200-17	PeproTech
Human serum albumin	0.1%	A9731	Sigma-Aldrich
Matrigel®	7.3-8.1 mg/mL	356234	Corning®
**Staining kit**			
ReadyProbes™ Cell Viability Imaging Kit (Blue/Red)		R37610	Invitrogen™
**40-plex, ELISA kits, and reagents**			
Bio-Plex Pro™ Human Chemokine Panel kit		171AK99MR2	Bio-Rad Laboratories Inc.
Human CXCL1/GRO-α DuoSet ELISA		DY275	R&D Systems
Human IL-8/CXCL8 DuoSet ELISA		DY208	R&D Systems
Human CXCL11/I-TAC DuoSet ELISA		DY672	R&D Systems
Human Lipocalin-2/NGAL DuoSet ELISA		DY1757	R&D Systems
3,3',5,5' tetramethylbenzidine substrate set		421101	BioLegend®
2N sulfuric acid stop solution		231-639-5	VWR
**RNA isolation and RT-qPCR reagents**			

RNeasy® Mini Kit		74106	Qiagen	
High-Capacity RNA-to-cDNA™ Kit		4387406	Applied Biosystems	
PerfeCTa SYBR Green FastMix, ROX		95073-012	Quantabio	
**Primers**		**Gene Globe ID**		
β-actin - Hs_ACTB_2_SG		QT01680476		
HO-1 - Hs_HMOX1_1_SG		QT00092645		
SOD2 - Hs_SOD2_1_SG		QT01008693		
LCN2 - Hs_LCN2_1_SG		QT00028098		
**Western blot reagents**				
Pierce BCA protein assay kit		23225	Thermo Scientific	
Cell Recovery Solution		354253	Corning®	
1x Complete® EDTA-free protease inhibitor	1X	11836170001	Roche Life Science Products	
NP-40	1%	492018	Sigma-Aldrich	
Phosphatase inhibitor cocktail 2	1X	P5726	Sigma-Aldrich	
Phosphatase inhibitor cocktail 3	1X	P0044	Sigma-Aldrich	
PageRuler™ Prestained Protein Ladder		26616	ThermoFisher	
NuPage™ 4-12% Bis-Tris gels		NP0321BOX or WG1402BX10	Invitrogen	
NuPAGE™ MOPS SDS running buffer	1X	NP0001	Invitrogen	
NuPAGE™ lithium dodecyl sulfate sample buffer	1X	NP0007	Invitrogen	
Trans-Blot Turbo nitrocellulose membrane 0.2 µm		1704158	Bio-Rad Laboratories Inc.	
Blocking Buffer for fluorescent Western blot		MB-070	Rockland Immunochemicals	
**Antibody**	**Dilution Factor**	**Catalog Number**	**Manufacturer**	**Lot Number**
NRF2 rabbit pAb	1:300	16396-1-AP #RRID:AB_2782956	Proteintech	00128335
HO-1 rabbit pAb	1:1000	10701-1-AP #RRID:AB_2118685	Proteintech	00119951

SOD2 mouse mAb	1:20000	66474-1-Ig #RRID:AB_2881840	Proteintech	10004881
p65 rabbit pAb	1:1000	A301-824A #RRID:AB_1264341	Bethyl	2
LCN2/NGAL rabbit pAb	1:2000	44058 #RRID:AB_2799257	Cell Signaling	1
GAPDH rabbit pAb	1:1000	5174 #RRID:AB_561053	Cell Signaling	8
β-actin rabbit mAb	1:20000	81115-1-RR #RRID:AB_2687938	Proteintech	23002493
Goat anti-Rabbit IgG (H&L) Secondary Antibody DyLight™ 800 Conjugated	1:5000	SA535571 #RRID:AB_2556775	Invitrogen	WK340127
Goat anti-Mouse IgG (H&L) DyLight™ 680 Conjugated	1:5000	35518 #RRID:AB_614942	Invitrogen	TL277851
**Softwares**				
MPM 6		Bio-Rad Laboratories Inc.		
StepOne, v2.3		Applied Biosystems #RRID:SCR_023455		
LI-COR Odyssey Fc Imager, v3.0.25		LI-COR Biotechnology #RRID:SCR_023227		
Image Lab # RRID:SCR_014210		Bio-Rad Laboratories Inc.		
GraphPad Prism 10		GraphPad Software Inc. #RRID:SCR_002798		
Rstudio, v4.4.2		#RRID:SCR_000432		
